# The Rlign algorithm for enhanced electrocardiogram analysis through heart rate–corrected ECG alignment for explainable classification and clustering

**DOI:** 10.1093/ehjdh/ztag067

**Published:** 2026-04-29

**Authors:** Lucas Plagwitz, Lucas Bickmann, Michael Fujarski, Alexander Brenner, Warnes Gobalakrishnan, Lars Eckardt, Antonius Büscher, Julian Varghese

**Affiliations:** Institute of Medical Informatics, University of Münster, Albert-Schweitzer-Campus 1/Building A11,Münster 48149, Germany; Institute of Medical Data Science, Otto-von-Guericke University Magdeburg, Leipziger Str. 44/Building 2,Magdeburg 39120, Germany; Institute of Medical Informatics, University of Münster, Albert-Schweitzer-Campus 1/Building A11,Münster 48149, Germany; Institute of Medical Informatics, University of Münster, Albert-Schweitzer-Campus 1/Building A11,Münster 48149, Germany; Institute of Medical Informatics, University of Münster, Albert-Schweitzer-Campus 1/Building A11,Münster 48149, Germany; Clinic for Cardiology II: Electrophysiology, University Hospital Münster, Albert-Schweitzer-Campus 1/Building A1,Münster 48149, Germany; Institute of Medical Informatics, University of Münster, Albert-Schweitzer-Campus 1/Building A11,Münster 48149, Germany; Clinic for Cardiology II: Electrophysiology, University Hospital Münster, Albert-Schweitzer-Campus 1/Building A1,Münster 48149, Germany; Institute of Medical Data Science, Otto-von-Guericke University Magdeburg, Leipziger Str. 44/Building 2,Magdeburg 39120, Germany

**Keywords:** Health informatics, Electrocardiogram, Machine learning, Signal processing

## Abstract

**Aims:**

Electrocardiogram (ECG) recordings are fundamental for diagnosing cardiac conditions. Recent advances in automatic ECG analysis have been dominated by deep learning, particularly convolutional neural networks (CNNs). CNNs excel in processing high-dimensional signal data, where their ability to automatically extract complex features has enabled significant progress. However, while CNNs are powerful for biomedical signal analysis, their application to ECG data also carries disadvantages, such as the requirement for large annotated datasets and limited explainability. To address these challenges, we aim to reintroduce shallow learning methods, such as linear classifiers, by leveraging the cyclic nature of ECG signals.

**Methods and results:**

We developed an adaptive transformation that restructures ECG signals into a fully structured format suitable for shallow learning algorithms. This method aligns R-peaks across all signals in a dataset and resamples the inter-QRS segments to match a predefined reference heart rate. The approach was systematically evaluated across tasks including classification, clustering, and explainability. Our transformation substantially improved the performance of shallow learning techniques. Compared with CNN approaches, shallow models trained on transformed ECGs achieved superior accuracy and interpretability in data-limited scenarios.

**Conclusion:**

We demonstrate that shallow machine learning methods, when combined with our alignment-based transformation, can reach CNN level performance in ECG analysis, especially under conditions of limited training data. This approach offers clear advantages in classification, clustering, and explainability and provides an accessible alternative to deep learning. To facilitate adoption and further research, we release a publicly available framework for ECG signal alignment at https://github.com/imi-ms/rlign.

## Graphical Abstract

**Graphical Abstract:**
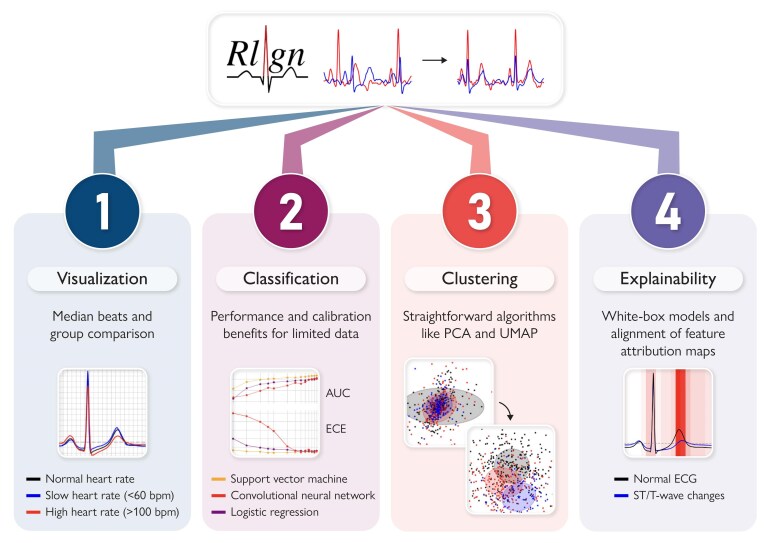

What’s new?

- We propose a novel R-peak alignment and reference heart rate–based resampling method for ECG transformation.
- Shallow models trained on transformed ECGs overcome CNN limitations in data demand and explainability.
- The transformation enables robust classification, clustering, and interpretability in ECG analysis.
- An open-source framework is released at https://github.com/imi-ms/rlign.

## Introduction

Machine learning has revolutionized the field of medical signal processing, offering unprecedented insights and advancements in the diagnosis, monitoring, and treatment of various health conditions.^1^ Among the different types of medical signals, the electrocardiogram (ECG) has gained substantial attention due to its high availability and standardization in signal acquisition and storage, and thus the availability of large datasets. ECGs, which record the electrical activity of the heart, are pivotal in diagnosing a variety of cardiac conditions. The ability to accurately analyse ECG data is, therefore, of paramount importance in cardiovascular medicine, making it a prime candidate for the application of machine learning techniques.

Major obstacles in the automatic analysis of ECG time series are variances in heart rates and temporal displacements of QRS complexes across different recordings. Although parameters such as heart rate variability may have some prognostic implications for the individual patient,^2^ the temporal dispersion of unsynchronized waveforms between different ECG recordings complicates their statistical and chronological comparison. Shallow learning methods, therefore, rely on prior feature extraction from the raw ECG signal.^3^ They can only learn statistical relationships within a set of predefined features that typically represent established intervals, amplitudes, or slopes around the classical ECG components during the cardiac cycle (e.g. *P*-wave, QRS complex, and T-wave).

Convolutional neural networks (CNNs) have emerged as a powerful tool for handling ECG data, thanks to their inherent ability to learn spatial hierarchies of features directly from the raw input signals.^4,5^ These learned features are independent of variations in heart rate and temporal offset, making CNNs highly effective for raw ECG analysis. However, CNNs are computationally expensive, require large numbers of positive examples, and are associated with low explainability.^6–9^ The complexity and non-transparent nature of CNNs can obscure the clinical decision-making process, making it difficult for clinicians to understand and trust the results.

In this paper, we introduce the ECG alignment algorithm Rlign designed to synchronize the temporal variations across ECG recordings. It is equipped with a heart rate–corrected alignment strategy that specifically adjusts for heart rate–dependent variations in PQ- and QT-intervals. We show how such R-peak alignment enhances the performance of supervised shallow learning models like support vector machines (SVM) and logistic regression (LR), and how the alignment facilitates clustering of ECG time series, overcoming the challenges posed by unaligned data, where time-domain clusters are obscured by temporal misalignments of cardiac cycles. Finally, we demonstrate how Rlign enhances explainability by aggregating feature attribution maps from Integrated Gradients across an entire dataset, rather than limiting the analysis to individual ECGs.

## Methods

### R-peak alignment pipeline

**Figure 1:**
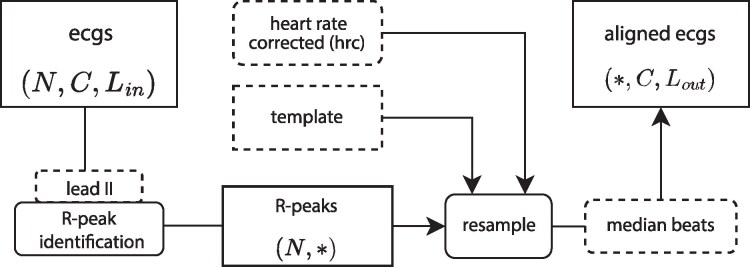
Schematic representation of the ECG alignment process. The diagram illustrates data in angular boxes and algorithms in rounded boxes, with dashed lines illustrating configurable settings. Key steps involve R-peak identification, correcting for heart rate variations, and resampling according to a standardized template. The final outputs are ECGs aligned at the R-peak, available as either a single median beat or the full continuous signal.

The alignment comprises two main steps: initially detecting R-peaks and subsequently resampling to the form of a predetermined uniform ‘template’. The entire process is schematically visualized in *Figure 1*. Our framework aligns with Scikit-Learn API conventions^10^ and features an efficient multiprocessing pipeline for rapid processing of multiple ECGs. To ensure uniformity across all ECGs, a standardized template is necessary. This template provides a consistent structure to which all signals are adjusted through transformation. It is defined by the recording duration, sampling frequency, desired beats-per-minute (bpm), and an initial offset. Both the recording duration and sampling frequency must align with those of the input ECGs. The number of QRS complexes is influenced by the total duration of the ECG and the chosen target bpm. The initial offset determines the starting position of the first QRS complex. In summary, the template essentially specifies the locations of R-peaks and the intervals between them.

#### R-peak detection and resampling

R-peak detection is based on the Python package NeuroKit2.^11^ As a preprocessing step, a single ECG lead (default lead II, user-configurable) is cleaned with a 0.5 Hz high-pass butterworth filter in fifth-order, followed by a powerline filtering in line and the detection algorithm.^12,13^ We employ NeuroKit2’s default method, which identifies QRS complexes by analysing the steepness of the absolute gradient and subsequently detects R-peaks as the local maxima within these regions.

Once R-peaks are identified on the reference lead and desired positions are established, the transformation process is applied uniformly across all leads. In this context, each ECG cycle is individually resampled to fit the target form through Fast-Fourier-Transform-based resampling provided by the Python package SciPy.^14^ Our tool provides three possible output formats:^1^ (i) a full-length signal resampled according to a template,^2^ (ii) a median beat representation,^3^ and (iii) a processed or unprocessed list view of all detected ECG cycles. Two distinct resampling algorithms are available, both align R-peaks but differ in their specific approaches. The linear strategy takes a complete ECG cycle and transforms it into the specified unified length. A tailored heart rate–corrected (hrc) strategy adjusts two heart rate–related intervals,


$$\vec{P_{onset}QRS_{onset}}(hr)=\left[R_{\;peak}-\frac{-0.351*hr+176.7+35}{1000*60/hr}*RR(hr),\;R_{\;peak}-35ms\right]$$

and


$$\vec{QRS_{offset}T_{offset}}(hr)=\left[R_{\;peak}+55ms,R_{\;peak}+\frac{230+170*60/hr-35}{1000*60/hr}*RR(hr)\right],$$

surrounding the QRS-complex from the observed heart rate to the target heart rate. The estimation of P-onset and T-offset is based on R-peak timing and heart rate, following delineation rules defined in prior studies.^15,16^ The coefficients preceding RR(hr) (distance between R-peaks) serve as relative scale factors for the dynamic adjustment of the PQ and ST intervals. Consequently, with increasing heart rate (hr), the scale factor increases. Notably, while these intervals are expressed in milliseconds (ms), they are influenced by the user-defined sampling rate, which determines the actual number of samples representing each interval. A fixed window spanning 35 ms before and 55 ms after the R-peak was used to represent the QRS complex, reflecting a heart rate–independent interval.

The aligned ECGs correspond to the specified template. Optionally, beats can be aggregated as representative waveform by computing the pointwise median across multiple consecutive ECG cycles. Furthermore, more advanced aggregation strategies are supported, such as querying a list view of unprocessed beats for independent inspection and processing. Intentionally, the entire process is optimized for multiprocessing, facilitating rapid processing of multiple ECGs.

### Evaluation and experiments

To explore the potential of R-peak alignment as a preprocessing step, a systematic assessment of its impact on both supervised and unsupervised machine learning was conducted. Additionally, the potential of explaining model decisions for SVM and CNN based classification approaches was investigated.

#### Simulation data

All analyses are based on the PTB-XL ECG dataset,^17^ a publicly available electrocardiography dataset, in version 1.0.3. The dataset contains 21,799 12-lead resting ECGs (10 s period with a 500 Hz sampling rate) from 18 869 patients. All data were annotated by up to two cardiologists. Therefore, ECGs can be grouped by characteristics or abnormalities. A total of 71 different ECG statements conform to the SCP-ECG standard and cover diagnostics. For most of our analyses, the five diagnostic superclasses were considered: ‘normal ECGs’ (NORM), ‘Myocardial Infarction’ (MI), ‘Conduction Disturbance’ (CD), ‘ST-T changes’ (STTC), and ‘Hypertrophy’ (HYP). For evaluation of classification performance, additional subcategories were considered, such as rhythm, form, and subclasses, following a procedure comparable to that described in prior studies.^18,19^ Since the PTB-XL dataset comes with 10 predefined folds (nine for training and one as a test set), these splits were used for all tasks. Our approach is evaluated alongside the median beats by the University of Glasgow ECG Analysis Program (Uni-G) and GE Healthcare Marquette 12SL ECG Analysis Program, as benchmarks in commercial state-of-the-art ECG analysis. Both benchmarks and the associated software outputs were obtained from the PTB-XL+ repository on Physionet.^20,21^

#### Classification performance and calibration

To assess the performance of time-sensitive predictive algorithms, two shallow learning models (LR and SVM) were evaluated against a CNN architecture optimized for time series classification, XceptionTime.^22^ Models were evaluated across four categories (rhythm, form, subclasses, and superclasses) using different training dataset sizes. Furthermore, an in-depth analysis of classification performance and model calibration for the diagnostic superclasses was conducted by reviewing learning curves generated from predefined training folds, starting with one-quarter of a fold and gradually incorporating all available folds (analogous to the methods in ^23^) allowing for a methodical comparison between the raw time series and hrc-realigned median beat representations. The primary performance metric was macro-averaged area under the receiver operating characteristic curve (AUC) for classification performance and expected calibration error (ECE) for calibration. LR and SVM models were obtained from the Scikit-Learn library; the CNN models were implemented using the PyTorch-based framework tsai.^10,24^ The default configuration was employed for all experiments, with no optimization of hyperparameters.

#### Model explainability

Global feature importance was evaluated using interval-wise permutation importance,^25^ as well as CNN-based attribution values through integrated gradients (IG).^26^ To compare the importance maps derived from these methods, they were applied to median beats in two distinct manners.

##### Permutation importance

An SVM was trained using the mean-standard-deviation-scaled median beats from the pre-defined training folds of the PTB-XL dataset. Nineteen intervals were organized, each consisting of 25 consecutive samples. Then, all features within each interval were shuffled and the effect on the test performance was assessed and compared with the original test set. This permutation process was conducted 20 times per interval. The alteration in performance was evaluated using the macro AUC. A large discrepancy between original performance and the performance based on shuffled input features indicates that the respective feature group had a key influence on the model output.

##### Integrated gradients

Attribution maps for the CNN models were generated with integrated gradients. Initially, binary classification models were trained on the training set. For these models, absolute IG maps were computed for all test set ECGs using the Python package captum.^27^ IG calculates the gradient of the model's output with respect to the input features by integrating the gradients along a straight path from a baseline to the actual input. The baseline reference for this method was set to the default of zero values. Through the application of R-peak alignment, attribution maps were transformed from their original space to an R-peak aligned median beat space. This temporal alignment permitted a global aggregation of these maps by averaging across all dataset instances.

Due to the inherent difficulty in directly evaluating the quality and plausibility of these methodologies, our analysis specifically emphasized abnormalities indicating specific regions in the ECG, notably STTC and non-acute MI (corresponding to stage ≥ II in the PTB-XL annotations). Non-acute MI cases were emphasized due to their association with changes in the QRS complex, which are expected to be identifiable from maps with STTC, predominantly linked to the T-wave. The correlation of all importance maps was assessed using Pearson's correlation coefficient.

#### Unsupervised machine learning

To demonstrate the capability of visualizing results from unsupervised analyses, two versions of principal components analysis (PCA) were applied to the data from the NORM, STTC, and non-acute MI subgroups. PCA was performed on various types of data, including raw signals, 12SL representations, Rlign linear median beats, and Rlign hrc beats. Additionally, for the aligned data, an interval-based PCA method targeted at explainable components axes was introduced. This method involves performing two separate PCAs, one for the QRS-complex and one for the T-wave. For each ECG segment, the respective interval is projected onto the first principal component, resulting in a single scalar score. The specific interval start and end points were determined by the pre-defined ECG template. Consequently, each heartbeat is represented by a two-dimensional feature vector, consisting of the first principal component scores of the QRS complex and the T-wave.

## Results

### Impact of heart rate on various resampling strategies for median beats

**Figure 2:**
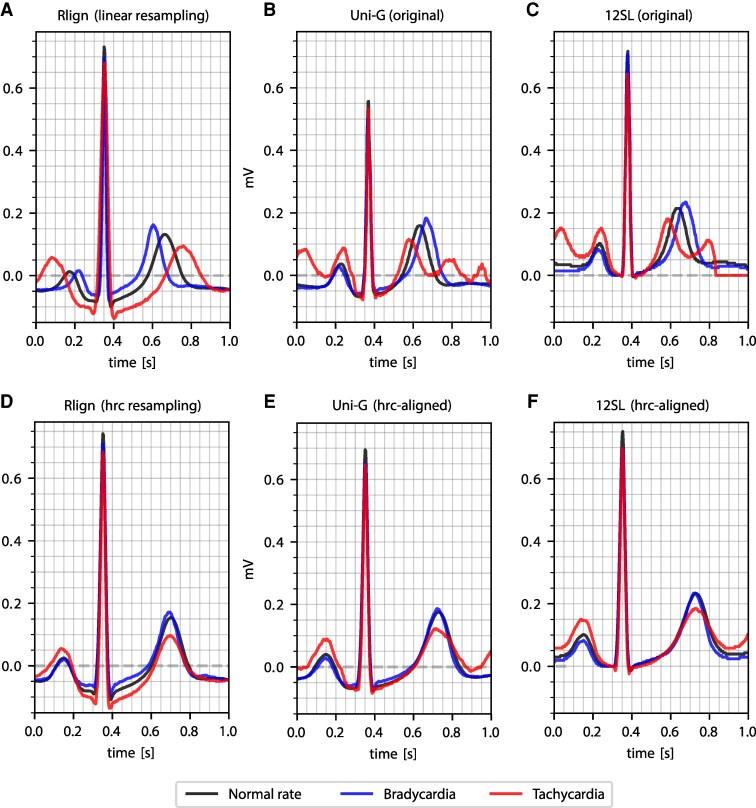
Summary of various median beat techniques and their impact on heart rate categories. Each chart displays three groups: normal rate (60–100 bpm), bradycardia (<60 bpm), and tachycardia (>100 bpm). Panel (*A*) represents Rlign with linear resampling, centred around the R-peak illustrating over-correction of heart rate dependent PQ- and QT alterations. Panel (*B*) displays the original output of group-specific point-wise median beats generated using the commercial Uni-G software. Panel (*C*) represents the original output of 12SL median beats, centred around the R-peak. Uni-G and 12SL retain the original signal sampling and do not achieve complete heart beat alignment, which is evident from the lower amplitude. Panels d-f illustrate our novel approach to resampling with adjustments for heart rate (heart rate correction, hrc), maintaining R-peak centring.

Utilizing the PTB-XL ECG dataset and its annotations, we computed median beats (pointwise median of all signals in one group) for ECGs labelled as normal (NORM), which were further grouped into bradycardia, normal rate, and tachycardia, following the PTB-XL rhythm definitions (e.g. SBRAD and STACH), to assess how different heart rate categories influence median beat representation. In *Figure 2*, we compare our Rlign algorithm for median beat calculation (*Figure 2A*) with the commercially available Uni-G (*Figure 2B*) and 12SL algorithms (*Figure 2C*), and additionally illustrate the influence of hrc on all three algorithms (*Figure 2D–F*). The rationale behind hrc resampling stems from the observation that PQ and QT intervals vary with heart rate, decreasing as heart rate increases. In the absence of appropriate corrections, an increasing heart rate would result in these intervals appearing disproportionately prolonged relative to the duration of the QRS complex in an aligned heartbeat. Because linear resampling stretches or compresses the entire cardiac cycle into a fixed number of samples, this phenomenon is evident in the linearly resampled median beats (*Figure 2A*), where the intervals are longest for tachycardia ECGs, followed by normal ECGs, and shortest for bradycardia ECGs. In principle, all three median-beat representations can be post-processed by hrc to improve comparability across ECGs with different heart rates. Since the Uni-G and 12SL algorithms do not include heart rate–related adjustments in their original output, PQ and QT intervals are preserved from the original input ECGs and are therefore displaced when comparing ECGs with different heart rates (*Figure 2B* and *C*). Moreover, a characteristic of both algorithms is the inclusion of more surrounding ECG signals around the single beat, which may influence machine learning model decisions. For example, in tachycardia ECGs, the presence of unaligned T-waves from the preceding beat and P-waves from the following beat could confound the analysis.

Conversely, our hrc demonstrates superior P-wave and T-wave alignment (*Figure 2E* and *F*). This approach ensures that the intervals are appropriately scaled relative to the QRS complex and mitigates the influence of adjacent cardiac activity on median beat calculation. This allows direct comparison of all ECG segments including the T wave, regardless of the underlying ECG heart rate.

### Classification performance and calibration

To investigate the influence of R-peak alignment on classification tasks, we assessed the performance of two shallow learning models—LR and SVM—against a well-established multi-channel deep learning technique for time series: the CNN architecture XceptionTime.^22^ Models were trained to predict the diagnostic classes provided in the PTB-XL dataset. For model training, we used three different inputs, which include (i) raw ECG data for the CNN, (ii) median beats calculated by the commercial Uni-G and 12SL software, and (iii) Rlign aligned ECG signals with hrc resampling in single median beat formats. The predictive performance of the different approaches is summarized in *Table 1*, with the macro AUC as the primary performance metric. Our analyses demonstrate that aligned data improved performance compared with the CNN baseline in the case of small training data. Across all classification categories, the combination of alignment with an SVM achieved the highest performance at training ratios of 1% and 10%. At a training ratio of 100%, the randomly initialized CNN matched the SVM performance, though the CNN maintained a distinct advantage in specific sub-tasks, most notably in rhythm classification. The clearest gain associated with our Rlign technique was observed for logistic regression, whereas SVM performance appeared comparable between Rlign, 12SL, and Uni-G alignment.

**Table 1:** Benchmark of ECG classification performance across alignment strategies

Method	PTBXL-Rhythm			PTBXL-Sub			PTBXL-Form			PTBXL-Super			Average		
Train Ratio	1%	10%	100%	1%	10%	100%	1%	10%	100%	1%	10%	100%	1%	10%	100%
UniG-LR	61.76	67.87	74	62.47	67.11	75.27	54.96	57.09	65.49	70.92	75.03	82.91	62.53	66.78	74.42
UniG-SVM	**66.76**	**75.76**	78.71	68.62	81.29	84.77	53.47	66.94	75.32	82.79	87.52	90.38	67.91	**77.88**	82.29
12SL-LR	62.97	72.47	83.32	67.21	69.60	76.59	55.56	60.98	65.30	72.61	78.03	83.62	64.59	70.27	77.21
12SL-SVM	60.93	71.49	76.01	75.25	**81.77**	**85.26**	57.02	65.40	73.51	83.23	**88.17**	**90.60**	**69.11**	76.71	81.35
Rlign-LR	59.08	65.97	79.22	69.83	72.97	82.64	56.66	**68.42**	**75.61**	77.33	83.89	86.02	65.73	72.97	80.87
Rlign-SVM	63.11	69.93	78.12	**74.29**	**80.87**	84.64	51.12	62.09	75.28	**83.84**	87.99	90.48	68.09	75.33	82.12
XceptionTime	60.49	67.01	**87.44**	63.52	68.77	82.16	57.01	59.64	73.02	69.99	83.9	88.49	62.75	69.83	82.78

ECG classification performance reported as macro AUC in one-vs.-rest scenarios across different transformation approaches and supervised learning models. The Rlign heart rate–corrected median beat alignment strategy is compared with Uni-G and 12SL median heartbeats using two shallow learning algorithms [logistic regression (LR) and support vector machine (SVM)] and with the CNN architecture XceptionTime applied to raw signals as a reference for state-of-the-art performance. Evaluations are based on the PTB-XL dataset across diagnostic superclasses, rhythm, subclass, and form categories.

**Figure 3:**
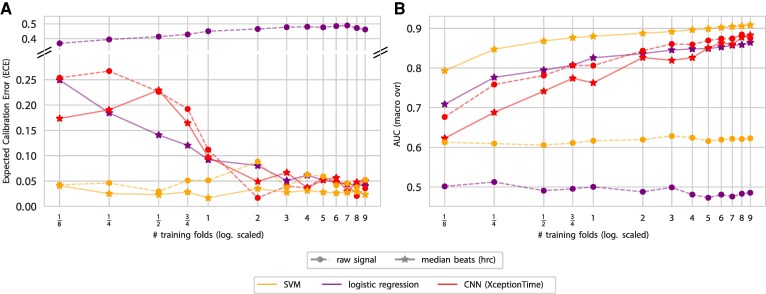
Rlign-specific analysis of classification performance and model calibration as a function of training data volume. This figure evaluates the impact of heart beat alignment by comparing raw ECG signals with R-aligned median beats. The comparison is made using three distinct models: linear regression, support vector machine, and the CNN architecture XceptionTime. (*A*) Multi-class calibration; (*B*) multi-class classification performance. In both panels, a single training fold consists of 1640 ECGs (Norm: 898, MI: 262, STTC: 251, CD: 174, HYP: 55).

To further test the influence of different sample sizes on classification performance and model calibration, we used the predefined cross-validation folds of the PTB-XL database. We trained models on 1/8, 1/4, 1/2, 3/4, 1, and multiples up to 9 folds, using the 10th fold for testing. Each single fold represented 1640 ECGs (Norm: 898, MI: 262, STTC: 251, CD: 174, HYP: 55). We assessed AUC for multiclass classification performance of the superclasses and ECE for model calibration. Results are displayed in *Figure 3*.

Without alignment, both LR and SVM models did not achieve meaningful predictive performance above the level of chance across all evaluated dataset sizes, indicating their limitations in handling unaligned data. In contrast, the CNN demonstrated classification capabilities also with unaligned data, exhibiting a near-linear relationship between predictive performance and dataset size on the logarithmic scale. However, on aligned data, the SVM model displayed superior predictive performance across the entire spectrum of dataset sizes, even outperforming the CNN. Similarly, LR exhibited enhanced performance with aligned data, albeit being outperformed by the CNN at larger dataset sizes. Interestingly, R-peak alignment did not improve the performance of the CNN; rather, it showed a minor trend towards decreased efficacy.

The analysis of model calibration revealed an advantage for both shallow learning algorithms over the CNN with limited training data. The ECE, which quantifies the difference between predicted probabilities and actual outcomes (lower is better), for the CNN model exceeds 0.2 with half a fold of training data, whereas LR and SVM maintain an ECE below 0.05. This lower ECE indicates more reliable and accurate probability predictions from LR and SVM in the presence of limited training data. With three or more training folds, the calibration errors of all algorithms level out. In Supplementary material online, *S1.1*, we provide a detailed analysis of model calibration for the Norm vs. STTC classification using a single fold of training data.

### Effects of R-peak alignment on unsupervised machine learning

**Figure 4:**
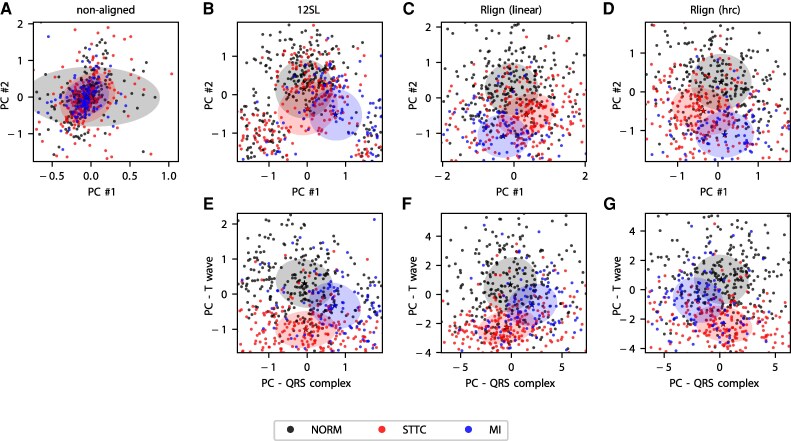
The realignment of ECGs enables principal component analysis (PCA) to project ECG data into a separable non-uniform two-dimensional space. The data set includes ECGs from the superclasses ‘normal ECGs’ (NORM), ‘ST-/T changes’ (STTC), and ‘Myocardial Infarction’ (MI, non-acute). The effect of various data transformation steps is depicted in a grid layout, with each column representing different alignment methods and each row showing different feature subsections. In panels (*A–D*), the full time series of all 12 leads were analysed, whereas panels e-g demonstrate the projection of the 12-lead QRS complex and T-wave onto their respective first principal components to form a two-dimensional feature vector.

The PCA performed on unaligned raw data resulted in a complete overlap of the diagnostic superclasses, as depicted in *Figure 4A*. This overlap signifies that PCA, without prior alignment of the ECG data, fails to represent features representative for the NORM, STTC, and non-acute MI superclasses. The distribution of all three classes around a common mean suggests that the variance captured by the principal components does not correlate with the clinically relevant differences among these diagnostic classes. When applying PCA to median beats calculated by the 12SL software, a separation between the superclasses NORM, MI, and STTC begins to emerge, as shown in *Figure 4B*. However, the class-wise means remain located within each other’s covariance ellipses. In contrast, with linear- or hrc-resampled signals processed through the Rlign algorithm, all superclasses diverge from one another in their principal components, as illustrated in *Figure 4C* and *D*. Since the linear and hrc-resampling approaches yielded comparable results, the observed improvement over 12SL may be primarily attributable to the incorporation of additional signal information from neighbouring ECG cycles.

Further analysis focused on differentiating the principal components associated with distinct compartments of the ECG, specifically the QRS complex and the T-wave (*Figure 4E–G*). Across all three median beat calculations, a consistent pattern emerges: normal ECGs and ST-T changes primarily differ in the T-wave component. In contrast, non-acute MI shows smaller divergence in the T-wave component but a clear separation in the QRS component, aligning with clinical expectations. Overall, the compartment-specific PCA reveals a high degree of agreement between the three approaches.

### Model explainability

**Figure 5:**
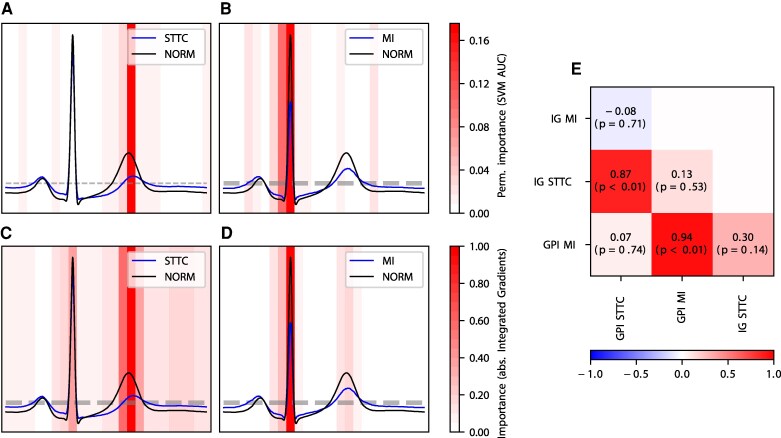
Importance maps and correlation for binary classification in STTC vs. NORM and MI (non-acute) vs. NORM. Panels (*A–B*) illustrate the use of a Grouped Permutation Importance (GPI) method, wherein 19 intervals undergo random permutation to assess their effect on SVM prediction in terms of AUC. Panels (*C–D*) display feature attribution maps obtained from the absolute values of integrated gradients (IG) using an XceptionTime CNN architecture. This architecture underwent training and examination on the unprocessed signal (limited to lead II), with the attribution scores aligned during a subsequent processing phase. For visualization, a median beat is shown for lead II. Intervals with highlighted colours are crucial for the overall prediction. Colormaps are depicted per row. (*E*) The correlation matrix visualizes the correlation across GPI and IG for NORM vs. STTC and NORM vs. MI (non-acute).

To further demonstrate the advantages of R-peak alignment to improve the explainability of machine learning models for ECG analysis, we conducted two exemplary binary classification tasks: ‘Normal ECG’ vs. ‘Myocardial Infarction’ (non-acute MI) and ‘normal ECG’ vs. ‘ST-T Changes’ (STTC). For each task, both an SVM and a CNN were trained. The relationship between input signal and output prediction was evaluated for both architectures using importance maps. *Figure 5A* and *B* illustrates the local permutation importance, where various intervals of the ECG signal undergo random permutation. This process assesses their impact on the SVM's predictions, with the AUC serving as the performance metric. For STTC prediction, the permutation importance peaks in the T-wave area, aligning with clinical expectations that ST-T changes are predominantly reflected in this segment. Similarly, for MI (non-acute), the highest importance is observed around the QRS complex, correlating with clinical expectations that non-acute MI exhibit distinct QRS changes on surface ECGs. *Figure 5C* and *D* represents a similar analysis for the CNN, employing integrated gradients (IG) to measure signal importance. Notably, the CNN, despite being trained on unprocessed raw data, prioritizes the same ECG compartments for prediction as the SVM trained on Rlign aligned signals. The T-wave region is deemed most critical for STTC prediction, while the QRS complex is highlighted for MI, underscoring the models’ alignment with clinical expectations. This qualitative consistency in importance across different models can be quantitatively validated through correlation analysis (*Figure 5E*). A high correlation is observed between the IGs for STTC prediction and the global permutation importance (GPI) for STTC but not with the GPI or IGs for MI predictions. Conversely, both importance metrics for MI show a high correlation, but not with the importances related to STTC. This analysis reinforces the reliability and clinical relevance of the importance metrics derived from both SVM and CNN models.

A notable innovation in our approach is the application of R-peak alignment to enable aggregated importance analyses over an entire dataset for CNNs. Typically, integrated gradients (IG) are calculated only for individual ECGs, limiting global explainability. By initially calculating individual IG maps for the entire test set and subsequently transforming these maps from their original space to the R-peak aligned space, we facilitate global aggregation of importance metrics across all dataset instances. This process underscores the utility of R-peak alignment in enhancing model explainability by providing a novel method for interpreting CNN decisions on a dataset-wide scale.

### Acceleration through multiprocessing

**Figure 6:**
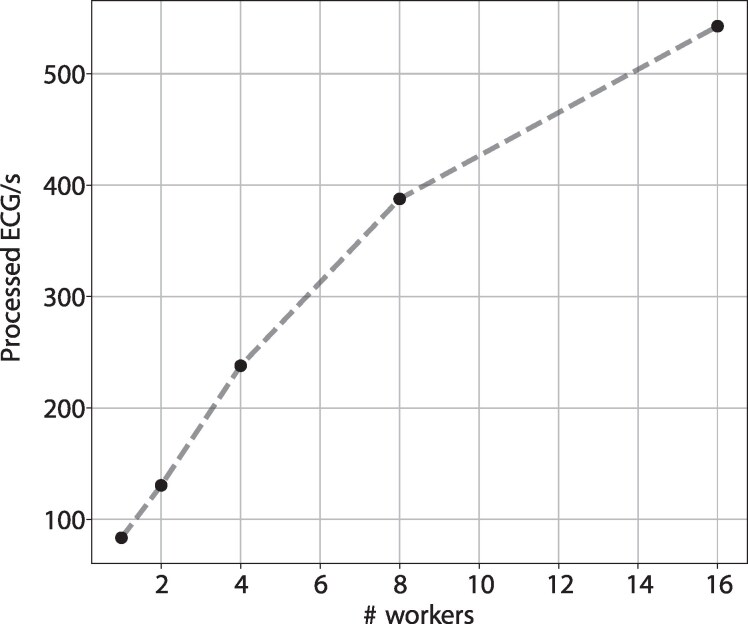
Acceleration through multiprocessing. This graph displays the reduction in alignment transformation time as the number of parallel workers increases from single to 2, 4, 8, 16. These tests were performed with Python 3.12 on an AMD EPYC 7543 32-core processor, utilizing resting-state ECGs (12 leads, 10 s, at a sampling rate of 500) as the input data.

The use of multiprocessing within the R-peak alignment pipeline significantly reduced processing times. *Figure 6* delineates the scalability of our algorithm, showcasing a reduction in processing time as the number of workers increases.

## Discussion

This study introduces the novel open-source ECG alignment algorithm Rlign, which synchronizes temporal variations across ECG recordings. The utilization of Rlign for heart beat alignment addresses a critical challenge in ECG analysis: the variance in heart rates and temporal displacements of QRS complexes. By aligning ECG recordings, our approach enables the application of feature-based ML models, such as SVM and LR, which traditionally lag behind deep learning models like CNNs in handling raw, unprocessed ECG data. In contrast to linear resampling, which stretches and compresses cardiac intervals uniformly and can particularly affect the PQ and ST intervals, Rlign applies heart rate correction in a more targeted manner. The alignment process facilitated by Rlign not only enhances the performance in classification tasks but also contributes to their calibration and explainability. This is crucial in clinical settings, where trust in, and understanding of the rationale behind a diagnostic prediction is as important as the prediction's accuracy itself.

Our results show that tabular models, when using R-peak alignment, perform better in classification and calibration than convolutional neural networks (CNNs) when there is only a limited amount of training data available. This is a significant finding, as acquiring a sufficient amount of training cases can be challenging. However, even with enough training data, the SVM demonstrates comparable performance to the CNN. This suggests that, for specific diagnostic tasks within ECG analysis, simpler ML models can achieve comparable performance to CNNs, provided that the input data is appropriately preprocessed and aligned.

Furthermore, the impact of R-peak alignment on clustering and unsupervised learning tasks highlights the importance of data preprocessing in uncovering inherent data structures obscured by temporal misalignments. The improved outcomes of unsupervised algorithms such as PCA on aligned data highlight the potential of R-peak alignment to enhance ECG data interpretation and identify clinically relevant patterns without the need for prior labelling. In this context, linear algorithms such as LR benefit particularly from hrc-resampling and, in our evaluations, were able to further improve the performance of established approaches such as Uni-G and 12SL (see *Figure 2* and Supplementary material online, section 1.3). This may largely be because warping signals to a fixed template eliminates morphological variances caused strictly by heart rate fluctuations, allowing the tabular-based models to focus on underlying cardiac pathology.

In terms of clinical application, the enhanced explainability of ML models achieved through R-peak alignment is a crucial and perhaps the most relevant advancement. The ability to aggregate importance metrics across an entire dataset, as demonstrated with CNNs, offers a new avenue for understanding model decisions on a broader scale. This capability is particularly valuable in a clinical context, where it can provide insights into the model's diagnostic ‘reasoning’, potentially uncovering novel diagnostic markers or patterns. Successful implementation of the Rlign algorithm for dataset wide feature importance analysis for CNNs has already been shown for classification and regression tasks.^28,29^ It should be noted that the accompanying category-level attention maps and median beats are aligned to the R-peak and are therefore intended to provide illustrative context for the importance distributions rather than detailed morphological interpretation. Due to inter-patient heterogeneity and opposing patterns, aggregation at the category level may lead to a reduced or attenuated apparent signal.

Despite the presented advantages, our study also acknowledges the limitations and challenges associated with the presented preprocessing of ECG data. For instance, while R-peak alignment improves the performance of shallow models, it does not significantly enhance the performance of CNNs, even when training data are limited. This observation may suggest that CNNs’ inherent feature extraction capabilities can sometimes negate the benefits of explicit alignment, indicating that the optimal preprocessing strategy may vary depending on the ML model being used. A critical aspect of ECG alignment is the potential for introducing bias into the signal that is not inherent in the data. Another limitation of Rlign is that heart rate compensation preserves a fixed interval around the R peak. This design was chosen because incorporating QRS delineation points into the compensation procedure could introduce an additional source of instability. Consequently, ECGs with unusually short or prolonged QRS complexes, such as paediatric ECGs or bundle branch block patterns, may not be optimally represented by this approach. Additionally, the alignment process or median beat calculation may filter out crucial information, such as variations between heartbeats or the presence of premature ventricular contractions, which can be essential for certain diagnostic applications. This limitation applies to any median-beat-based approach, and even if we demonstrate reconstruction fidelity comparable to established methods (see Supplementary material online, *S1.2*), these informational losses are unavoidably suppressed by the median-beat representation. Consequently, median beats should not be interpreted as faithful surrogates of the underlying rhythm without contextual information, such as heart rate or beat-level dynamics. For applications where rhythm information or individual-case interpretability is critical, a non-aggregated, beat-by-beat analysis constitutes the more appropriate choice, which is also supported by Rlign.

In general, the necessity of suitable time-based alignment strategies or time-invariant methods to enable biomedical signal analysis is well established. In the past, various strategies have been deployed to address the challenges posed by temporal variations within ECG recordings. Historically, alignment strategies have primarily focused on the temporal positioning of the QRS complex, given its significance in representing ventricular depolarization and its prominence in the ECG waveform. Early methods, as described by Escalona *et al*. (1993),^30^ introduced QRS alignment through fixed signal points within a bandpass-filtered segment of the ECG, aiming to enhance the signal's clarity for subsequent high-frequency analysis. This approach demonstrated the advantage of alignment in improving the signal-to-noise ratio and facilitating more accurate detection of cardiac events. Further research by Shaw and Savard (1995)^31^ expanded on this concept by employing alignment methods to study beat-to-beat variations. Their work highlighted the critical role of alignment in enabling the analysis of ECG signal variations for diagnosing and monitoring cardiac abnormalities. The impact of alignment strategies on the application of deep neural networks has been shown by Xu *et al*. (2019),^32^ who demonstrated that pre-aligned data could improve network performance by up to 10%. However, our study did not reproduce the finding of improved CNN accuracy with pre-aligned data. This discrepancy may be due to differences in alignment strategies as well as the use of a more complex network architecture, which may be better designed to mitigate overfitting and may have influenced the results.

Moreover, the use of ECG alignment for visualizing abnormalities has been a critical application, aiding clinicians in the interpretation of median beats and facilitating a more intuitive understanding of patient-specific cardiac activity. The work of Al-Zaiti *et al*. (2023)^33^ refers to the use of commercial software like the Philips DXL diagnostic algorithm for alignment and feature extraction, indicating the clinical relevance and utility of these techniques. However, the proprietary nature of such tools and the lack of open-source alternatives have posed significant barriers to accessibility and widespread adoption in research and clinical settings. In response to these accessibility barriers, we propose Rlign as an open-source framework for ECG alignment. By building upon the foundation laid by previous research, Rlign offers a robust and transparent methodology, facilitating the application of both traditional and machine learning-based analysis techniques. In contrast to earlier methods, Rlign is designed to be adaptable, efficient, and easily integrated into existing ECG analysis pipelines, representing a significant advancement in the field.

Another essential consideration in the application of machine learning models, particularly in the medical field, is the computational cost and power efficiency of these algorithms. Shallow learning models, such as LR, are not only computationally less demanding but also significantly more power-efficient compared with their deep learning counterparts. This difference in power consumption is critically important when deploying ML models in resource-constrained environments, such as implantable medical devices like implantable cardioverter defibrillators or event recorders. These devices operate under stringent power constraints, as maximizing battery life is essential to reduce the need for surgical replacements. The introduction of any machine learning algorithm into such devices is expected to increase computational demand, potentially shortening battery life due to the added power consumption required for processing. However, our hope with the introduction of novel preprocessing algorithms like Rlign is to mitigate the extent to which battery life is shortened. Rlign's efficiency in aligning ECG signals enables the use of less computationally demanding models for ECG analysis. The relative simplicity and lower computational requirements of these shallow models are expected to lessen the impact on battery life compared with more complex algorithms like CNNs.

In conclusion, the hrc median beats provide a robust overview of the signal, which can be effectively interpreted by shallow learning systems such as LR and SVM. This type of signal allows the application of standard dimensionality reduction techniques and clustering methods, facilitating unlabelled pattern recognition. Moreover, CNN architectures can benefit from Rlign by aligning feature attribution maps, enhancing explainability from local to global scales. Given these capabilities, Rlign stands out as a versatile tool that not only improves the transparency but also boosts the performance of various machine learning models in ECG analysis. Further research is needed to validate our findings in broader benchmarks and independent datasets.

## Supplementary Material

ztag067_Supplementary_Data

## Data Availability

All simulations are based on the publicly accessible PTB-XL data set.^17^ The Rlign source code is available under the terms of the MIT license at GitHub (https://github.com/imi-ms/rlign).
