# Supplementary material for: The Rlign algorithm for enhanced electrocardiogram analysis through heart rate–corrected ECG alignment for explainable classification and clustering
Source: Eur Heart J Digit Health. 2026 Apr 29;7(5):ztag067. doi: 10.1093/ehjdh/ztag067 (PMC13184624; doi:10.1093/ehjdh/ztag067)
Supplement: ztag067_Supplementary_Data [file ztag067_supplementary_data.pdf]

Supplementary Material to

The Rlign Algorithm for Enhanced Electrocardiogram Analysis through Heart-Rate-Corrected ECG Alignment for Explainable Classification and Clustering

Lucas Plagwitz, M.Sc.[1,*], Lucas Bickmann, M.Sc.[2,*], Michael Fujarski, M.Sc.[1], Alexander Brenner, M.Sc.[1], Warnes Gobalakrishnan, B.Sc.[1], Lars Eckardt, M.D.[3], Antonius Büscher, M.D.[1,3,*], Julian Varghese, M.D.[2,*]

[1] Institute of Medical Informatics, University of Münster, Albert-Schweitzer-Campus 1 / Building A11, 48149 Münster, Germany

[2] Institute of Medical Data Science, Otto-von-Guericke University Magdeburg, Leipziger Str. 44 / Building 2, 39120 Magdeburg, Germany

[3] Clinic for Cardiology II: Electrophysiology, University Hospital Münster, Albert-Schweitzer-Campus 1 / Building A1, 48149 Münster, Germany

[*] These authors contribute equally to this work.

Corresponding author:

Dr. med. Antonius Büscher

University Hospital Münster

Albert-Schweitzer-Campus 1, 48149 Münster, Germany

Tel. 0251 83 58380

E-Mail: antonius.buescher@ukmuenster.de

1. Supplementary Methods

1.1. Calibration

In the medical field, attributes such as good calibration are gaining importance alongside the pure performance of machine learning models. To evaluate a model's calibration, we follow the definition provided by Guo et al. (1). Specifically, we divide the possible confidence interval [0, 1] into M bins of size $\frac{1}{M}$. Let Bm represent the set of sample indices where the prediction confidence falls within the interval $I_m = (\frac{m-1}{M}, \frac{m}{M}]$. The accuracy of Bm is defined by

$$acc(B_m) = \frac{1}{B_m} \sum_{i \in B_m} 1\,(\hat{y}_i = y_i)$$

where $\hat{y}_i$ and y_i are the predicted and true class labels for sample i. The average confidence within bin B_m is defined as

$$conf(B_m) = \frac{1}{B_m} \sum_{i \in B_m} \hat{p}_i$$

where $\hat{p}_i$ is the confidence for sample i. The expected calibration error (ECE) is given by

$$acc(B_m) = \frac{1}{B_m} \sum_{i \in B_m} \mathbf{1}\,(\hat{y}_i = y_i)$$

The expected calibration error (ECE) is given by

$$ECE = \sum_{m=1}^{M} \frac{|B_m|}{n} |acc(B_m) - conf(B_m)|,$$

where n is the number of samples. In the main text, the ECE loss was calculated using 10 bins, whereas Fig. S2 provides a clearer depiction using 5 bins. Logistic regression, a calibrated method by design, is often used as a base comparison. Fig. 3 shows a very stable SVM through the ECE, significantly more stable than the CNN, but also calibrated as the logistic regression. With increasing training data, the ECE difference vanishes. Fig. S2 shows the calibration of the models for one training fold in more detail. While the SVM is almost perfectly calibrated, the LR displays a slight shift in the second bin, where predictions in the 0.2-0.4 range are slightly overestimated. This contrasts with the CNN, which shows no prediction in the 0-0.2 range. Consequently, the first bin is entirely absent, leading to a severe underestimation in the first bin.

1.2. Morphological Fidelity of Reconstructed ECG Median Beats

To evaluate the fidelity of the reconstructed waveforms, we utilized the PTB-XL dataset and its associated PTB-XL+ extension. For each recording, the ground truth QRS complexes were derived from the raw 10-second signal (Lead II) by identifying R-peaks, extracting segments from -35ms to 55ms around the R-peak. Each unprocessed beat was compared against median beats generated by three distinct algorithms: our proposed Rlign (hrc) and two closed-source methods, Uni-G and the 12SL, provided via the PTB-XL+ feature set.

Prior to metric calculation, all waveforms were aligned via cross-correlation of R-peaks and min-max normalized to ensure scale invariance. The primary evaluation metric was the Root Mean Square Error (RMSE) between the signal derived plain median and the ground truth. To ensure a comprehensive evaluation, performance was stratified across multiple clinical dimensions, including patient demographics (age), signal characteristics (voltage amplitude), and pathological conditions (diagnostic super-classes and specific arrhythmia labels).

The comparative analysis indicates that Rlign achieves QRS morphological fidelity that is largely comparable to established commercial algorithms. As illustrated in Supplemental Figure 2, median RMSE values are closely aligned across methods and clinical dimensions, with only minor differences. Across age groups, increasing age is associated with higher RMSE and variance for all methods, suggesting a shared sensitivity of alignment performance to age-related ECG morphology rather than algorithm-specific effects. A similar pattern is observed for signal amplitude, where all algorithms show increased error and dispersion in low-voltage recordings (≤1 mV), consistent with reduced signal-to-noise ratios. Across diagnostic categories, all methods show low median RMSE for normal sinus rhythm, ST/T changes, and hypertrophy, whereas myocardial infarction and conduction disturbances exhibit higher RMSE and variance across all methods. Arrhythmic recordings further increase variability due to beat-to-beat irregularity, particularly in supraventricular arrhythmia and atrial fibrillation. Importantly, these dimensions are interrelated: myocardial infarction and conduction disturbances occur more frequently in older patients and are often associated with altered amplitudes and more complex morphologies. The observed increase in RMSE and variance therefore appears to reflect ECG characteristics rather than algorithmic differences.

Overall, similar trends are observed across age, amplitude, and diagnostic groups, and QRS reconstruction fidelity remains broadly comparable among all evaluated algorithms. This convergence points to a methodological gap, suggesting that current median-beat alignment strategies share common limitations.

1.3. Heart-rate-correction for state-of-the-art median-beat algorithms

The median beats derived from Uni-G and 12SL can be retrospectively resampled following the heart-rate-correction formulas, at least for the segments spanning from P-onset to QRS-onset and from QRS-offset to T-offset. This procedure yields a visualization that closely resembles our Rlign alignment strategy. Figure 2 (e-f) illustrates the heart-rate-corrected median beats of Uni-G and 12SL, averaged across the PTB-XL rhythm groups sinus rhythm, sinus tachycardia, and sinus bradycardia. The transformation facilitates improved temporal alignment of the P- and T-waves across methods. However, differences in the R-peak morphology remain clearly visible between Uni-G and 12SL. Importantly, this alignment has a beneficial impact on linear analysis methods such as principal component analysis (PCA). As shown in Supplementary Figure 3, the resulting representations emphasize pathological variations, such as myocardial infarction (MI) and ST-T changes (STTC), while reducing the influence of heart-rate-related variability.

Supplementary References

1. Guo C, Pleiss G, Sun Y, Weinberger KQ. On calibration of modern neural networks. In: Proceedings of the 34th International Conference on Machine Learning - Volume 70. Sydney, NSW, Australia: JMLR.org; 2017. p. 1321–30. (ICML'17).

2. Supplementary Figures

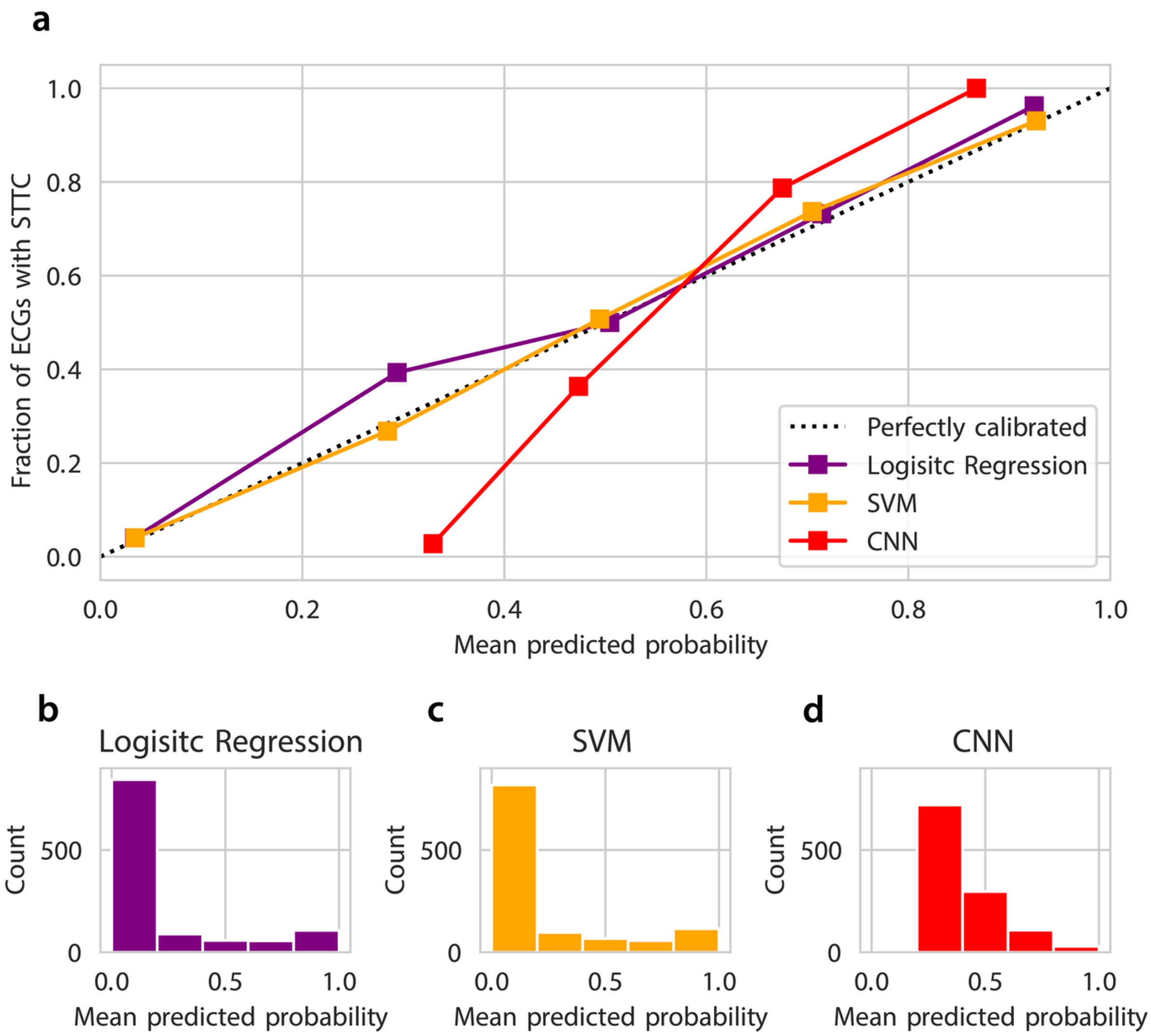

Supplementary Figure 1 The calibration of the three classification algorithms (hrc-resampled median beats for logistic regression, SVM, and raw data CNN) is shown for the special case of using 1 training fold in the binary distinction between norm and STTC. All predictions were divided into 5 bins of equal width.

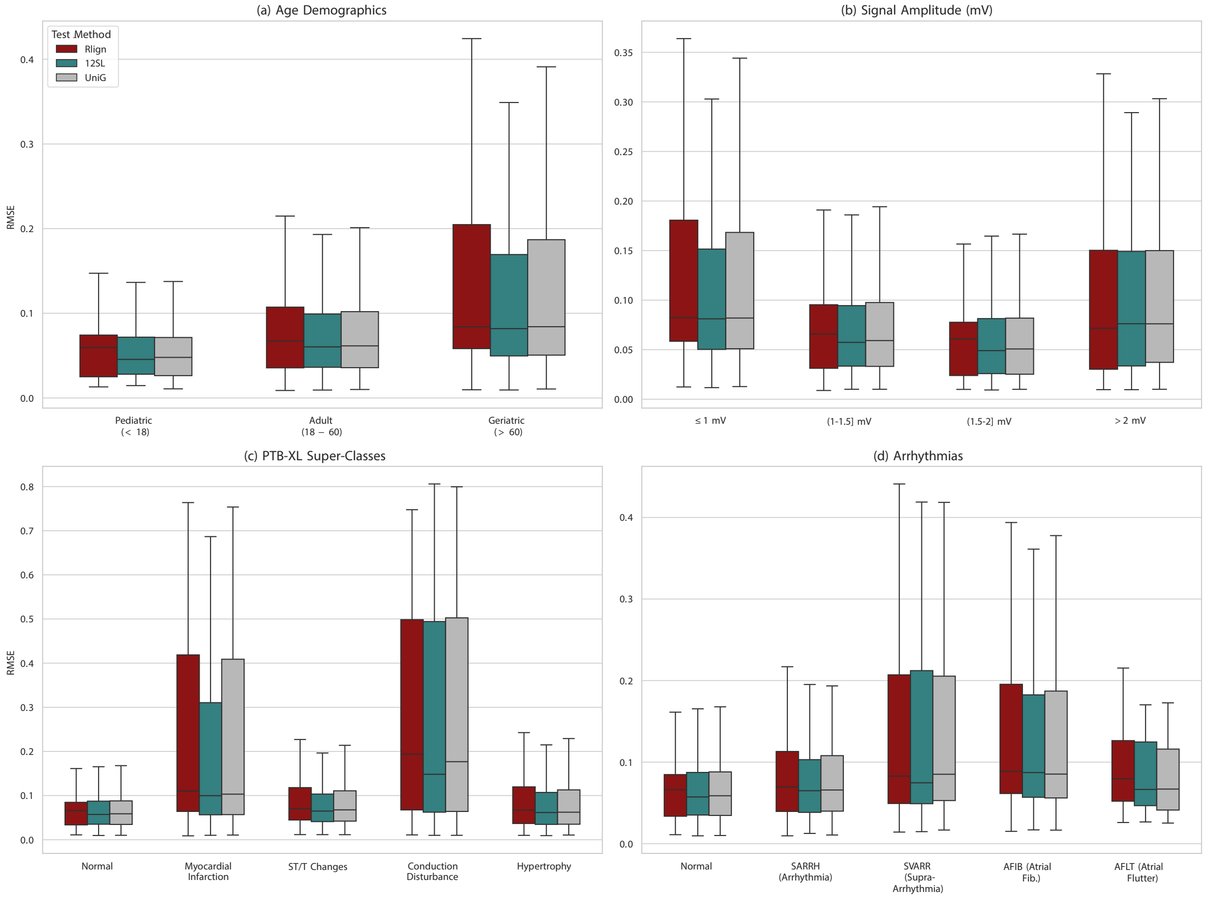

Supplementary Figure 2 Comparative assessment of morphological reconstruction error across clinical subgroups. The Root Mean Square Error (RMSE) between the generated median beat and the ground-truth unprocessed beats is visualized for three algorithms: the proposed Rlign (hrc) (red), 12SL (teal), and Uni-G (grey). Results are stratified by (a) patient age group, (b) peak-to-peak signal amplitude (voltage), (c) diagnostic superclasses, and (d) arrhythmia types.

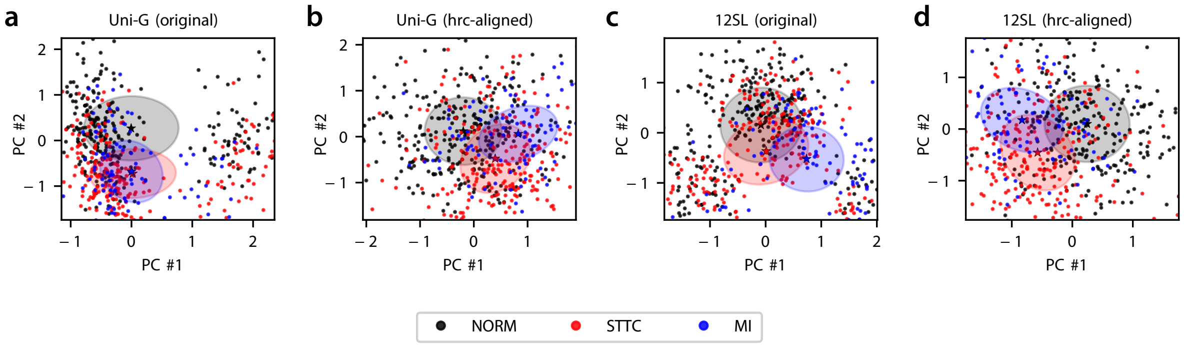

Supplementary Figure 3 Effect of the heart-rate-correction (hrc)-based resampling strategy on the PCA analysis, shown in parallel to the corresponding figure in the main manuscript. Results are shown for median beats derived from the Uni-G and 12SL algorithms.
